# Patterns of Avoiding Nutrition Mistakes in Metropolitan Adolescents Are Associated with Sex, Nutrition Knowledge, Physical Activity, and Family Environment

**DOI:** 10.3390/nu13020433

**Published:** 2021-01-29

**Authors:** Małgorzata Drywień, Magdalena Górnicka, Stanisław Kulik, Krzysztof Górnicki

**Affiliations:** 1 Department of Human Nutrition, Institute of Human Nutrition Sciences, Warsaw University of Life Sciences—SGGW, 159C Nowoursynowska Str., 02-776 Warsaw, Poland; malgorzata_drywien@sggw.edu.pl (M.D.); stankulik@o2.pl (S.K.); 2 Department of Fundamental Engineering and Energetics, Institute of Mechanical Engineering, Warsaw University of Life Sciences—SGGW, 164 Nowoursynowska Str., 02-787 Warsaw, Poland; krzysztof_gornicki@sggw.edu.pl

**Keywords:** eating habits, food, lifestyle, physical activity in leisure time, nutrition knowledge dietary pattern, parental education, siblings, high school students, metropolis

## Abstract

A comprehensive approach to the identification of the relationship between behaviors limiting nutrition mistakes, nutrition knowledge, and physical activity in the context of the family environment has not yet been widely explored. We aim to identify patterns of avoiding nutrition mistakes in high school students from Warsaw, Poland, and to assess their associations with nutrition knowledge (NK), physical activity (PA), body mass index (BMI), demographic, and family environment characteristics. A cross-sectional study involving 616 high school students, aged 16–19, was conducted. The data were collected by distributing questionnaires. The k-means method was used for cluster analysis, and logistic regression was used to assess the adherence to identified patterns. We identified three patterns: Prudent Ones (45%), Inconsequent (39%), and Rebels (16%). About 70% of adolescents had insufficient NK. The adherence to the Rebels pattern was lower by 85 % in girls, by 68% in students with younger siblings, and was about 4.0-times higher in children of mothers with primary education, 2.4 times higher in students with insufficient NK, and 1.9-times higher in students living in a family with more than 4 persons. The groups of adolescents with feature characteristics of the Rebels and Inconsequent are possible targets for intervention and require further in-depth research to explain their lack of attempts to avoid nutrition mistakes. The results clearly indicate the necessity of including metropolitan teenage boys in effective nutritional education for the rationalization of their dietary behavior.

## 1. Introduction

Adequate nutrition in adolescence is important for both present and future well-being because this period is important for preventing the consequences of nutritional deficiencies and excesses [1,2]. The diet of adolescents is not properly balanced, and the disproportions between the various nutrients are significant and have been observed in different countries [3,4,5,6]. Adolescence is a key period for the development of lifestyle and eating behaviors, which puts this group at a particularly high risk of obesity and being overweight. Unhealthy nutritional behaviors are common during adolescence and include excessive consumption of sweetened beverages, sweets, fat, and/or salty snacks, combined with reduced consumption of fruits, vegetables, milk, and dairy products [7]. These eating habits are associated with an increased risk of obesity and cardiovascular disease, which is why monitoring them is crucial because they are thought to last a lifetime [8,9]. A low-quality diet can also be caused by skipping breakfast or replacing lunch with snacks, often reported among adolescents [10]. As a result, there is an insufficient nutrient intake, which, in the case of low physical activity, increases the risk of obesity and being overweight [9,11].

Nutritional behaviors are shaped from childhood and are often modified during adolescence. They depend on a variety of factors, including sex, sociodemographic conditions, the family environment, and peers. Nutritional behaviors have a direct impact on the physical development, health, and identity of adolescents [12,13]. Behaviors such as avoiding alcohol, snacking, or eating breakfast have a direct impact on the health of adolescents, contributing to its improvement and, thus, to the achievement of better academic performance [10,14]. Considering that adolescents gain nutrition knowledge from the media, school programs, or educational campaigns, they may be aware of some of the nutrition mistakes they are making and their health consequences. The nutrition knowledge of adolescents is rather inadequate and selective [15], which is why educational programs should be specially adapted to this age group [16]. It may be hypothesized that adolescents know the relationship between health and diet and may try to avoid nutrition mistakes, such as eating nonrecommended foods and unhealthy eating behavior. However, this behavior is related to sociodemographics, BMI, physical activity, and nutrition knowledge.

Therefore, the aim of this study is to identify patterns of avoiding nutrition mistakes and to assess their association with sex, BMI, characteristics of the family environment, physical activity in leisure time, and nutrition knowledge among high school students from Warsaw.

## 2. Materials and Methods

### 2.1. Study Design & Data Collection

**Figure 1:**
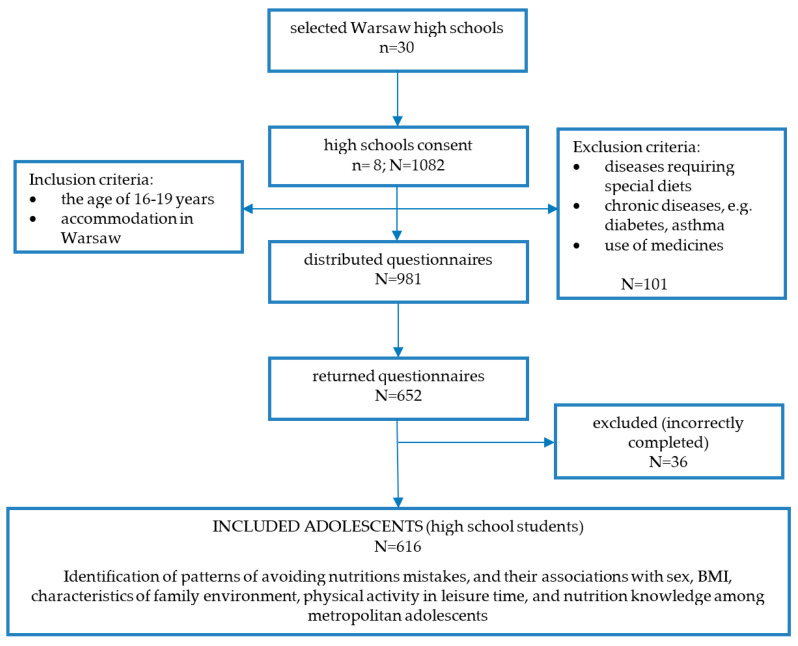
Flowchart: study design and data collection (n—number of schools; N—number of high school students).

The cross-sectional study was conducted on high school students between Autumn 2014 and Spring 2015 (Figure 1). From 93 Warsaw public high schools, 30 schools with similar education profiles were selected. Schools with special education profiles (sport, bilingualism, arts) and organizational structures (school complex for adolescents in a wide age range) were excluded. The management of each school received an invitation to participate in the research study. The headmasters of eight high schools responded positively to the invitation (27% of the selected high schools). 

In each school, paper–pen questionnaires (*N* = 1082) were distributed in classes. The following inclusion criteria were used: the age of 16–19 years, living in Warsaw, a Polish metropolis (defined as a large urban center of high economic and social importance, hence the term “metropolitan high school students”). The following exclusion criteria were applied: diseases requiring special diets, chronic diseases, e.g., diabetes or asthma, use of medicines, and incorrectly completed questionnaires. All participants were informed about the necessary details and agreed to participate in the study. In the case of minors, written consent of the parent or guardian was obtained. A total of 981 students met the criteria (549 girls and 432 boys), but only a total of 652 students responded to the survey. Completed and returned questionnaires (*N* = 652) were verified by research staff, and incorrectly or incompletely filled questionnaires were excluded (*N* = 36). The final sample was comprised of 616 students (63% of the total sample; 34% of boys, 88% of girls). 

### 2.2. Measures

#### 2.2.1. Unhealthy Nutritional Behaviors and Patterns of Avoiding Nutrition Mistakes 

The selected questions from The Beliefs and Eating Habits Questionnaire (KomPAN), developed and validated by the Commission of Behavioral Determinants of Nutrition from the Polish Academy of Sciences (Warsaw, Poland) [17], were used to assess the frequency of consumption of not-recommended products and unhealthy behaviors in the past six months (Table S1, Supplementary Materials). The frequency of consumption of the following products was examined: sweets, fast food, fried meat, sweetened beverages, energy drinks, beer, and strong alcohol. The frequency of unhealthy nutritional behaviors, as in adding salt or sugar, was tested. The following numerical codes were applied: 1—(almost) never, 2—(almost) once a week, 3—1–2 times a week, 4—3–4 times a week, 5—every day, 6—several times a day. For unhealthy nutritional behaviors such as not eating breakfasts and snacking, the following numerical codes were applied: 1—(almost) never, 2—(almost) once a week, 3—several times a week, 4—every day. 

To assess the limitation of not-recommended product consumption (sweets, fast food, fried meat, sweetened beverages, energy drinks, alcohol) and the limitation of unhealthy behaviors (adding salt or sugar, snacking, night eating), four statements were used: (i) I do not limit myself; (ii) I am looking for a healthier alternative; (iii) I try to eat/drink this very rarely; (iv) I do not eat/drink/practice this. Due to the lack of existing measures, these items were tested in a pilot study on a group of 30 adolescents who did not take part in the main research. Thus, the statement “I do not eat/drink this” was added. The following categories and numerical codes were used for Statement (i)—not limiting (1); for Statements (ii) and (iii)—limiting (2); for Statement (iv)—not eating (3).

The data on both the frequency and limitation described above were used to identify patterns of avoiding nutrition mistakes. It was assumed that the most healthy eating pattern would be the one with less frequency, a willingness to not eat or to limit nonrecommended products, and a willingness to limit unhealthy eating behavior. The most unhealthy pattern was assumed to be eating with greater frequency and a willingness to consume nonrecommended products.

#### 2.2.2. Sociodemographics, Anthropometrics Variables

Data on sex, age, weight, height, and family environment characteristics such as household size, siblings (no siblings, younger or older siblings), parental education (primary, secondary, university), accommodation (house, flat), and household structure (living with one parent, with both parents, with parents and grandparents) were collected. Body mass index (BMI) was calculated using self-reported weight (kg) and height (m). For students aged 16–18 years, the following age-sex-specific BMI cut-offs and categories were applied: BMI < 18.5 kg/m^2^ is categorized as thinness; BMI = 18.5 to 24.9 kg/m^2^ is categorized as normal weight; BMI ≥ 25 kg/m^2^ is categorized as overweight and obesity [18]. For students aged 19, adult criteria and cut-offs were applied [19].

#### 2.2.3. Nutrition Knowledge

To assess nutrition knowledge, 8 questions from the test, developed and validated by the group of Polish experts, were selected [20]. These questions verified the obligatory knowledge included in the school curriculum and were accepted by the teachers who are responsible for the high school curriculum. The subjects were required to select a proper answer to the questions on the following subjects: the number of meals in a day; the daily amount of beverages required for an adolescent; the interval between the last meal and bedtime; restrictions on the consumption of products sweetened with aspartame; health reasons to consume a low-protein diet; a starchy food product; the richest source of vegetable protein; food product rich in dietary fiber (more details in the questionnaire in Section 2; Table S1, Supplementary Materials)). The correct answer was given a mark of 1, incorrect as 0; the maximum number of points was 8. For further analysis of the results, the subjects were divided into two groups: insufficient level (0–4 points) and sufficient level (5–8 points) of nutrition knowledge.

#### 2.2.4. Physical Activity in Leisure Time

The question about physical activity (PA) in leisure time is from The Beliefs and Eating Habits Questionnaire (KomPAN) [17]. The following categories were used: low PA—sitting lifestyle, TV viewing, reading books, light housework, 1–2 h a week; moderate PA—walking, cycling, gymnastics or other light physical activity performed 2–3 h a week; high PA—cycling, running, other sports activities requiring physical effort, performed over 3 h per week. The school program includes 4 h of sports activities per week; therefore, the level of physical activity in leisure time was crucial for the PA assessment. 

#### 2.2.5. Data Analysis

Categorical variables are presented as a sample percentage (%). Patterns of avoiding nutrition mistakes were derived using cluster analysis. The input variables were 22 data points of the frequency of consumption of not-recommended products, the frequency of unhealthy behaviors, and the limitation of consumption of not-recommended products and unhealthy behaviors. The k-means clustering algorithm, based on the Euclidean distances, was used. Three clusters were selected. The description of the cluster includes the following features: sex, age, BMI, family environment, level of physical activity in leisure time, and nutrition knowledge level. For the codes of numeric values, which refer to the frequency of consumption of not-recommended products, unhealthy nutritional behaviors, and behaviors limiting nutrition mistakes, mean and standard deviations were calculated, and an analysis of variance for comparing means of separate groups was used. 

Logistic regression modeling was applied to assess the adherence to identified patterns by demographic factors, nutrition knowledge, physical activity, and family characteristics. Odds ratios (ORs) and 95% confidence intervals (95% CIs) were calculated. The ORs were adjusted for sex, BMI, characteristics of family environment, nutrition knowledge, and physical activity, excluding the modeled variable from the confounders set, respectively. The significance of ORs was assessed by Wald’s statistics. For all tests, *p* < 0.05 was considered significant. Analyses were performed using IBM^®^ SPSS^®^ Statistics software (IBM Corp. Released 2017. IBM SPSS Statistics for Windows, ver. 25.0. Armonk, NY, USA: IBM Corp.). 

## 3. Results

### 3.1. Group and Cluster Characteristics

The majority of the subjects were girls (76%), students aged 16–17 years. The body mass index in the vast majority of participants indicated a normal weight. Almost 20% of students were thin, and only 8% had excessive body weight. Almost 70% of students had insufficient nutrition knowledge. Most of the participants in the study group lived in households with no more than four people (77%), in flats (61%), with both parents (69%). (Table 1).

In the study group, three clusters were identified, whose sociodemographic characteristics, PA level in leisure time, and nutrition knowledge are shown in Table 1.

### 3.2. Patterns of Avoiding Nutrition Mistakes and Their Determinants

Patterns of avoiding nutrition mistakes, derived using cluster analysis, were the following:

Cluster 1. (Prudent Ones: *I know and I limit*)—the most healthy eating pattern. This group includes subjects who, significantly, seldom consumed not-recommended products and snacks and most often limited or did not eat not-recommended products (Table 2). 

Cluster 2. (Inconsequent: *I know something and I limit sometimes*); this group includes students who most often added sugar and salt but limited most not-recommended products and unhealthy behaviors (Table 2). 

Cluster 3. (Rebels: *I do not know and I do not limit*)—the most unhealthy eating pattern. This group includes students with the highest frequency of consumption, who do not limit not-recommended products and snacking and rarely consume breakfast (Table 2). 

Girls (aOR = 6.34; 95% CI (3.89–10.34); *p* < 0.001) and students with moderate PA in leisure time (aOR = 2.12; 95% CI (1.14–3.94); *p* = 0.018) had over six- and two-times higher adherence to the Prudent Ones pattern (Table 3). The adherence was lower by 62% in students with younger and older siblings (aOR = 0.38; 95% CI (0.23–0.62); *p* < 0.001) and by 57% in students with insufficient nutrition knowledge (aOR = 0.43; 95% CI (0.29–0.62); *p* < 0.001).

The adherence to the Inconsequent pattern (Table 3) was about two-times higher in students with younger or older siblings (aOR = 1.97, 95% CI (1.02–3.82), *p* = 0.045 and aOR = 2.26, 95% CI (1.35–3.78), *p* = 0.002, respectively) and three-times higher in students with both younger and older siblings (aOR = 2.98; 95% CI (1.80–4.94); *p* < 0.001), 1.6 times higher in children of mothers with secondary education (aOR = 1.64; 95% CI (1.10–2.45); *p* = 0.016), and 1.5 times higher in students with insufficient nutrition knowledge (aOR = 1.50; 95% CI (1.03–2.16); *p* = 0.033).

The adherence to the Rebels pattern (Table 3) was lower by 85% in girls (aOR = 0.15; 95% CI (0.09–0.25); *p* < 0.001) and by 68% in students with younger siblings (aOR = 0.32; 95% CI (0.13–0.81); *p* = 0.015). There was almost four-times higher adherence in children of mothers with primary education (aOR = 3.83; 95% CI (1.67–8.75); *p* = 0.001), 1.85-times higher adherence in students living in families larger than 4 persons (aOR = 1.85; 95% CI (1.01–3.41); *p* = 0.047), and 2.35 times higher adherence in students with insufficient nutrition knowledge (aOR = 2.35; 95% CI (1.33–4.14); *p* = 0.003).

In the crude model, the adherence to the Prudent Ones pattern was 1.5 times higher in students living in flats (OR = 1.48; 95% CI (1.08–2.09); *p* = 0.017), while it was 49% lower in students with older siblings (OR = 0.51; 95% CI (0.32–0.81); *p* = 0.004); however, those associations became nonsignificant after the adjustment (Table 3).

## 4. Discussion

Among the three patterns of avoiding nutrition mistakes among high school students from a Polish metropolis, two opposite patterns were identified: the Prudent Ones pattern, with limited unhealthy behaviors (45%), and the Rebels pattern, with no limits to unhealthy behaviors (16%). As we hypothesized, the avoidance of nutrition mistakes was associated with sex, nutrition knowledge, physical activity, and family environment. Higher adherence to the Prudent Ones pattern was found in girls and students with moderate physical activity, while adherence was lower in students with siblings and students with insufficient nutrition knowledge. The adherence to the most unhealthy pattern (Rebels) was found in students with families larger than 4 persons, children of mothers with primary education, and students with insufficient nutrition knowledge. Having siblings and insufficient nutrition knowledge increased the chance of adherence to the Inconsequent pattern. An unexpected finding of the study was the lack of association of the identified patterns with BMI.

Our results indicated that sex was a significant determinant of the adherence to the identified patterns, and metropolitan girls were more likely to avoid nutrition mistakes. This confirms a strong association of the female gender with the most healthy pattern and the male gender with the most unhealthy pattern. This is consistent with previous results indicating that sex significantly determines the choice of food and eating behavior. Young women more often avoid high-fat foods, eat fruits and vegetables, and report the greater importance of healthy eating, so dietary choices depend on the perception of femininity and masculinity [21]. Women often report significantly more prohealth behaviors than men and show healthier lifestyle patterns [22]. The results of other studies have shown that boys are the main consumers of junk food, sweets, and sweetened drinks and girls less so [22,23,24,25].

Living in a household with more than 4 people was associated with higher adherence to the Rebels patern. This relationship is difficult to explain directly. There are models of home eating environments based on sociocognitive and socioeconomic theories that suggest that the influence of the family, including parenting practices and other aspects of home, will shape the adoption of healthy eating behavior [26,27]. The available data explain the relationship between the size and structure of the family and qualitative and quantitative nutrition [28] but do not explain the relationship with the motivation of adolescents to specific eating behaviors; this is a field for further research. Therefore, the explanation and description of the relationships between complex aspects of adolescent environments (family, home, peers) and dietary behaviors remain a challenge.

We found an association between patterns of avoiding nutrition mistakes and having siblings. Having siblings was strongly associated with higher adherence (by 97–98%) to the Inconsequent pattern, characterized by less frequent attempts to limit the consumption of sweets, sugar, and salt. The number and profile of siblings may be related to compliance with specific nutritional patterns or health-related markers, both positive and negative [29,30]. A Spanish study showed that having more than one sibling is associated with a higher nutritional risk [28]. In the research of Cook et al., cited by [29], persons having siblings showed lower consumption of all nutrients, with the exception of carbohydrates and added sugar, which was confirmed by our results. One possible explanation is that more siblings means that the family has less to spend on each family member [31]. The only child, especially if it is a girl, may have better economic and social conditions to follow a healthy lifestyle. However, it may also be related to the age and sex of the siblings. In our study, students with siblings had greater adherence to the Inconsequent pattern, and those with younger siblings had lower adherence to the Rebels pattern. The complexity of the adolescent–siblings–parents relationship and dietary behavior should be taken into account in further research for greater insight.

Our study also confirmed that the adherence to patterns other than the Prudent Ones pattern was associated with the lower education of mothers. It is consistent with van Ansem et al.’s results [32], which indicated that children of mothers with higher education were characterized by prohealth eating behaviors more often than children of mothers with lower education. Unhealthy foods were more available at home for adolescents of mothers with a low educational level. Bere et al. [33] found that adolescents’ fruit and vegetable consumption was linked to their mothers’ level of education, while Watts et al. [26] showed that high-fat food availability was lower in families where the mothers had higher education. As they are more responsible for food supply and choice, mothers are shown as a “factor” affecting and shaping children’s eating behaviors [34]. On the one hand, this result points out the importance of a mother’s education, but on the other, high school students who have achieved a certain degree of education and have greater experience will acquire the skills of formal, operational, and critical thinking towards norms and restrictions to which they often disagree [35] and oppose. Without a doubt, this association indirectly points out the role of the mother’s education and knowledge, but it is probably more important in the earlier stages of child development; this issue requires a longitudinal study.

Despite the presence of elements of nutrition sciences during school education, the majority of students were characterized by insufficient nutrition knowledge. This indicates the lack of effectiveness of the health program in improving the lifestyle of adolescents. Our study shows that high school students with insufficient nutrition knowledge have higher adherence to unhealthy patterns (Rebels or Inconsequent) and lower adherence to the most healthy pattern (Prudent Ones). Despite the fact that some authors have shown that nutrition knowledge has a weak correlation with actual food choices [36], successful health promotion should be a better fit for the needs, attitudes, and characteristics of the target group and should focus on increasing nutrition literacy (skills and behavior) during adolescence [37,38]. Our results highlight the importance of nutritional knowledge for prohealth behaviors of high school students. Additionally, higher adherence to the Prudent Ones pattern is associated with moderate physical activity in leisure time; the coexistence of physical activity and a healthy diet in adolescents [6,39,40] and in girls [41] has been confirmed in the literature.

In our study, patterns of avoiding nutrition mistakes were not associated with BMI. The explanation for the lack of such a relationship can be found in the specificity of the studied group, which mostly declared high physical activity. Additionally, the percentage of high school students who were thin was two-times more than those with excess body weight (17% vs. 8%). Furthermore, in our study, 20% of the girls and 10% of the boys were classified as thin. Thinness is more common among adolescent girls and young women in European countries such as the Czech Republic, Sweden, Greece, and France than among boys. Additionally, the prevalence of thinness among Polish children is higher than in other European countries or Australia and the USA. This tendency may be related to sociocultural conditions, the fashion for a slim figure created in the media, and greater weight control in adolescent girls. The authors also point to the role of psychological factors such as self-esteem, body image, and emotional state [42].

Our results show that Polish high school students from the metropolitan area made typical nutrition mistakes such as unrestricted eating of sweets, fast food, and fried meat, drinking sweetened beverages and energy and alcohol drinks, and also adding salt or sugar, snacking, and eating at night. Such behaviors are characteristic for this age group and are associated significantly with the adolescents’ peer behaviors [9,43,44,45,46,47]. This is a natural phenomenon, as adolescence is a period of independent decision-making and increased autonomy in personal choices, which include practicing unhealthy behaviors. The results of the conducted research showed that some of the adolescents were aware of their nutrition mistakes and tried to limit them, but most of them did not avoid nutrition mistakes. Bryan et al. [48] pointed out that persuading adolescents to change their eating behavior often does not bring the expected result because they face a behavioral obstacle in the form of a sense of their own autonomy. Adolescents make the right food choices when they have a sense of independence from adult control and associate these behaviors with improved social status [48]. Both modifiable determinants revealed in our study, physical activity and nutrition knowledge, should be used as the basis for the development of educational and public health promotion programs for adolescents. Future programs need to be better suited to the sociodemographic characteristics of the groups and should include changing adolescent lifestyles.

### Strength and Limitation of the Study

Our study had a number of strengths and limitations that should be mentioned. This study is novel due to its comprehensive approach, taking into account lifestyle behaviors, the level of nutrition knowledge, as well as family environment characteristics. A major strength is the relatively large sample of metropolitan adolescents. The study area is a metropolis, the capital city of Poland, so the results of this study may be a good basis for further research in high school students from big cities. This is due to the fact that after the completion of primary school, almost half of the graduates continue their education in high schools, of which over 28% are located in the 18 largest Polish cities [49]. Ideally, the sample would have included a larger number of boys. There was a lower response rate for boys vs. girls, which was taken into account by poststratification weights, but, in the future, alternative recruitment strategies to encourage boys and to reach groups that vary in sex are needed. Most nutrition studies suffer from selection bias, in which there are more females and individuals with more health/nutrition knowledge participating [50]. Additionally, a relatively lower percentage of students with excessive body weight and a higher percentage with high physical activity levels seem to confirm the existing tendency; it should be noted that data about body mass and height as well as physical activity were self-reported. However, the data on health-related behaviors obtained by self-reporting has been found to offer satisfying reliability [51].

This study was also limited by the cross-sectional design, which precludes the investigation of casual relationships. However, it provides data to more complex study designs or to create interventions targeted at improving health-related behaviors.

## 5. Conclusions

Our research revealed that avoidance and no-avoidance nutrition mistakes were associated with sociodemographics, PA in leisure time, and nutrition knowledge but not with BMI. Among the three identified nutrition-mistakes avoidance patterns, two opposite patterns were identified: the Prudent Ones pattern, with limited unhealthy behaviors, and the Rebels pattern, with no limitation to unhealthy behaviors.

Female gender, no siblings, nutrition knowledge, and greater PA in leisure time increased the adherence to the Prudent Ones pattern, which can be described as “know and avoid nutrition mistakes”. Having siblings, mother’s secondary education, and insufficient nutrition knowledge increased the adherence to the Inconsequent pattern, who “know something about healthy eating and sometimes avoid unhealthy behaviors”. Male gender, living in large families, insufficient nutrition knowledge, and mother’s primary education increased the adherence to the Rebels pattern, who “do not know and do not avoid unhealthy behaviors”.

From a public health perspective, implementing interventions that increase nutrition knowledge and physical activity in high school students are important for improving prohealth dietary habits. The groups of high school students with the feature characteristics of the Rebels and Inconsequent are possible targets for intervention. Further in-depth research is needed to explain their lack of attempts to avoid unhealthy behaviors.

## Figures and Tables

**Table 1:** Characteristics of the group and clusters.

Variables		Total *N* = 616		Prudent Ones *N* = 280	Inconsequent *N* = 238	Rebels *N* = 98
		*n*	%	%	%	%
Sex	boys	148	24	11	27	53
	girls	468	76	89	73	47
Age	16	218	36	37	34	37
	17	210	34	31	39	28
	18	156	25	26	22	32
	19	32	5	6	5	3
BMI	thinness	106	17	15	20	16
	normal	458	74	77	72	72
	overweight and obesity	52	8	8	8	12
Family environment characteristics						
Accommodation type	flat	376	61	66	61	47
	house	240	39	34	39	53
Household size	≤4	472	77	79	78	65
	>4	144	23	21	22	35
Household structure	with one parent	136	22	25	21	16
	with both parents	424	69	65	71	74
	with parents and grandparents	56	9	10	8	10
Siblings	none	132	21	28	15	18
	younger	224	36	31	42	37
	older	176	29	27	31	27
	both	84	14	14	12	18
Father’s education	primary school	108	17	17	20	14
	secondary	146	24	23	27	19
	higher	362	59	60	53	67
Mother’s education	primary school	36	6	6	4	10
	secondary	144	23	20	29	21
	higher	436	71	74	67	69
Nutrition knowledge	sufficient	194	31	41	25	19
	insufficient	422	69	59	75	81
PA level in leisure time	low	58	9	7	11	12
	moderate	112	18	16	18	27
	high	446	72	77	71	61

BMI, body mass index.

**Table 2:** Identified pattern characteristics (mean and standard deviation).

Variables	Clusters (*N* = 616)			
	Prudent Ones *n* = 280	Inconsequent *n* = 238	Rebels *n* = 98	*p* *
Frequency of consumption of not-recommended products ^1^				
sweets	3.7 ± 1.4 ^b^	4.5 ± 1.2 ^a^	4.7 ± 1.3 ^a^	<0.001
fast food	1.4 ± 0.6 ^c^	1.8 ± 0.7 ^a^	2.7 ± 1.3 ^b^	<0.001
fried meat	3.3 ± 1.2 ^a^	3.1 ± 1.3 ^a^	3.3 ± 1.2 ^a^	0.104
sweetened beverages	1.6 ± 0.8 ^c^	2.6 ± 1.2 ^a^	4.8 ± 1.2 ^b^	<0.001
energy drinks	1.3 ± 0.6 ^a^	1.3 ± 0.6 ^a^	2.2 ± 1.4 ^b^	<0.001
beer	1.6 ± 0.7 ^a^	1.6 ± 0.9 ^a^	2.5 ± 1.2 ^b^	<0.001
strong alcohol	1.3 ± 0.6 ^a^	1.4 ± 0.7 ^a^	1.9 ± 1.0 ^b^	<0.001
salt/adding salt	2.9 ± 1.6 ^c^	4.6 ± 1.2 ^a^	4.2 ± 1.4 ^b^	<0.001
sugar/adding sugar	1.9 ± 1.1 ^c^	5.2 ± 0.8 ^a^	3.6 ± 1.8 ^b^	<0.001
Frequency of behaviors ^2^				
eating breakfast	3.6 ± 0.8 ^a^	3.6± 0.8 ^a^	3.1 ± 1.1 ^b^	<0.001
snacking	2.8 ± 1.0 ^c^	3.1 ± 0.8 ^a^	3.5 ± 0.7 ^b^	<0.001
night eating	1.5 ± 0.8 ^a^	1.4 ± 0.8 ^a^	1.4 ± 0.9 ^a^	0.635
Limitation of consumption of not-recommended products and unhealthy behaviors ^3^				
sweets	2.0 ± 0.4 ^c^	1.7 ± 0.5 ^a^	1.4 ± 0.5 ^b^	<0.001
fast food	2.2 ± 0.5 ^c^	2.0 ± 0.6 ^a^	1.5 ± 0.5 ^b^	<0.001
fried meat	1.9 ± 0.5 ^c^	1.5 ± 0.5 ^a^	0.9 ± 0.5 ^b^	<0.001
sweetened beverages	2.4 ± 0.6 ^c^	1.9 ± 0.6 ^a^	1.3 ± 0.5 ^b^	<0.001
energy drinks	2.6 ± 0.6 ^a^	2.7 ± 0.6 ^a^	1.9 ± 0.8 ^b^	<0.001
alcohol in total	2.0 ± 0.7 ^a^	2.1 ± 0.7 ^a^	1.5 ± 0.7 ^b^	<0.001
adding salt	2.2 ± 0.8 ^c^	1.6 ± 0.8 ^a^	1.4 ± 0.8 ^b^	<0.001
adding sugar	2.3 ± 0.8 ^c^	1.4 ± 0.7 ^a^	1.9 ± 0.8 ^b^	<0.001
snacking	2.7 ± 0.7 ^c^	2.2 ± 1.0 ^a^	1.7 ± 0.9 ^b^	<0.001
night eating	2.9 ± 0.5 ^c^	2.5 ± 0.9 ^a^	2.2 ± 1.0 ^b^	<0.001

* ANOVA; ^1^ for the consumption of not-recommended products: 1—(almost) never, up to 6—several times a day; ^2^ for the frequency of selected nutritional behaviors: 1—(almost) never, up to 4—every day; ^3^ for limiting attempts: 1—not limiting, 2—limiting, 3—not eating/not drinking; ^a–c^ Tukey posthoc test; ^a–c^ the same letters indicate homogenous groups.

**Table 3:** Associations between patterns of avoiding nutrition mistakes with demographic factors, family characteristics, nutrition knowledge, and physical activity (odds ratios: crude and adjusted, 95% confidence intervals).

Variables	Prudent Ones		Inconsequent		Rebels	
	OR (95% CI) ^1^	aOR (95% CI) ^2^	OR (95% CI)	aOR (95% CI)	OR (95% CI)	aOR (95% CI)
Sex						
girls (ref. boys)	4.24 (3.17; 7.96)	6.34 (3.89; 10.44)	0.77 (0.50; 1.05)	0.67 (0.45; 1.00)	0.20 (0.13; 0.31)	0.15 (0.09; 0.25)
*p*	<0.001	<0.001	ns	ns	<0.001	<0.001
Household size						
>4 people (ref. ≤ 4 people)	0.78 (0.53; 1.14)	0.95 (0.59; 1.55)	0.86 (0.65; 1.34)	0.76 (0.48; 1.19)	1.95 (1.03; 2.62)	1.85 (1.01; 3.41)
*p*	ns	ns	ns	ns	0.038	0.047
Accommodation type						
flat (ref. house)	1.48 (1.08; 2.09)	1.22 (0.83; 1.79)	0.99 (0.61; 1.17)	0.96 (0.067; 1.37)	0.50 (0.44; 1.03)	0.75 (0.46; 1.22)
*p*	0.017	ns	ns	ns	ns	ns
Siblings						
younger (ref. none)	0.44 (0.35; 1.06)	0.95 (0.47; 1.89)	2.19 (0.99; 3.19)	1.97 (1.02; 3.82)	1.23 (0.48; 2.03)	0.32 (0.13; 0.81)
*p*	ns	ns	ns	0.045	ns	0.015
older (ref. none)	0.51 (0.32; 0.81)	0.61 (0.37; 1.03)	1.97 (1.34; 3.58)	2.26 (1.35; 3.78)	1.12 (0.52; 1.74)	0.59 (0.30; 1.17)
*p*	0.004	ns	0.002	0.002	ns	ns
both (ref. none)	0.56 (0.27; 0.66)	0.38 (0.23; 0.62)	1.32 (1.56; 4.00)	2.98 (1.80; 4.94)	1.71 (0.98; 2.12)	0.71 (0.36; 1.40)
*p*	<0.001	<0.001	<0.001	<0.001	ns	ns
Mother’s education						
primary (ref. higher)	0.93 (0.38; 1.54)	0.62 (0.29; 1.34)	0.64 (0.29; 1.33)	0.60 (0.28; 1.30)	2.02 (1.22; 5.29)	3.83 (1.67; 8.75)
*p*	ns	ns	ns	ns	0.014	0.001
secondary (ref. higher)	0.68 (0.52; 1.12)	0.65 (0.42; 1.00)	1.54 (1.05; 2.25)	1.64 (1.10; 2.45)	0.92 (0.42; 1.27)	0.76 0.42; 1.39)
*p*	ns	ns	0.026	0.016	ns	ns
Nutrition knowledge						
insufficient (ref. sufficient)	0.43 (0.30; 0.61)	0.43 (0.29; 0.62)	1.67 (1.12; 2.28)	1.50 (1.03; 2.16)	2.10 (1.32; 3.83)	2.35 (1.33; 4.14)
*p*	<0.001	<0.001	0.011	0.033	0.003	0.003
Physical activity in leisure time						
moderate (ref. low)	1.78 (0.92; 2.89)	2.12 (1.14; 3.94)	0.76 (0.40; 1.19)	0.64 (0.36; 1.15)	0.60 (0.40; 1.73)	0.53 (0.24; 1.16)
*p*	ns	0.018	ns	ns	ns	ns
high (ref. low)	1.24 (0.59; 2.21)	1.50 (0.73; 3.09)	0.75 (0.37; 1.31)	0.64 (0.33; 1.26)	0.92 (0.61; 3.26)	0.93 (0.39; 2.40)
*p*	ns	ns	ns	ns	ns	ns

^1^ Crude odds ratio (OR); ^2^ Odds ratios adjusted (aOR); 95% CI—confidence interval; *p* for Wald’s statistics; ns—not significant.

## Data Availability

The data presented in this study are available on request from Krzysztof Górnicki.

## Supplementary Materials

The following are available online at https://www.mdpi.com/2072-6643/13/2/433/s1, Table S1: Questionnaire (English version).

## Abbreviations

BMI	Body mass index
FFQ	Food frequency questionnaire
PA	Physical activity
