# Supplementary material for: Patterns of Avoiding Nutrition Mistakes in Metropolitan Adolescents Are Associated with Sex, Nutrition Knowledge, Physical Activity, and Family Environment
Source: Nutrients. 2021 Jan 29;13(2):433. doi: 10.3390/nu13020433 (PMC7911558; doi:10.3390/nu13020433)
Supplement: Supplementary file 1 [file nutrients-13-00433-s001.pdf]

Table S1. Questionnaire (English version)

Questions		Answers
**Section 1. Socio-demographic features**		
1.	Sex	Girl /Boy
2.	Age	…..
3.	Height [cm]	…..
4.	Weight [kg]	…..
5.	Number of people in the household	…..
6.	Father education	Primary/ secondary/ university
7.	Mother education	Primary/ secondary/ university
8.	Siblings	I have no siblings/ I have older siblings/ I have younger siblings/ I have older and younger siblings
9.	Family composition (I live with…)	One of the parents/ both parents/ parents and grandparents
10.	Type of accommodation	Flat/ house
11.	Frequency of extracurricular activities	1-2 hours a week (low PA – sitting lifestyle, TV viewing, reading books, light housework),/ 2-3 hours a week (moderate PA - walking, cycling, gymnastics or other light physical activity performed)/ performed over 3 hours per week (high PA - cycling, running, other sports activities requiring physical effort),
**Section 2. Nutritional education test**		
Your opinion about nutrition:		
1.	How many meals should be eaten during the day?	…… (4-5)*
2.	Daily consumption of beverages (water, coffee, tea, juices) by an adult should be approximately	0.5 litters/ 1 litter/ 1,5 litters/ 2,0 litters/ I don't know
3.	The last meal should be eaten no later than….. at bedtime	15 min/ 30 min/ 1 hour/ 2 hours/ I don't know
4.	Products sweetened with aspartame must not be consumer by children suffering from:	Galactosemia/ phenylketonuria/ hepatitis/ cystic fibrosis/ I don't know
5.	A low-protein diet is used in the case of:	Kidney failure/ extensive burns/ body wasting/ I don't know
6.	A food product rich in complex carbohydrate is:	Honey/apple/ buckwheat/ vegetable oil/ I don't know
7.	The richest source of vegetable protein among the listed is:	Buckwheat/ hazelnuts/ banana/ vegetable oil/ I don't know
8.	A food product rich in dietary fiber is:	Honey/ vegetable oil/ meat/ wholemeal bread/ I don't know<br>**the right answer underlined*
**Section 3. Food frequency consumption**		
While answering to the questions, please consider foods you have eaten in the past six months during your meal times, between the meals as well as those that have been eaten at home and out.		
	**How often do you eat:**<br>• Sweets<br>• Fast food (e.g. hamburgers, pizza, hot-dogs);	

• Fried meat<br>• Sweetened beverages (e.g. coca-cola, pepsi, sprite, fanta, lemonade)<br>• Energy drinks (e.g. red bull)<br>• Beer<br>• Strong alcohol<br>• Salt / adding salt<br>• Sugar / adding sugar	Never/ once a week/ 1-2 times a week/ 3-4 times a week/ every day/ several times a day
**How often do you:**<br>• Eat late in the evening (before going to bed)<br>• Eat at night<br>• Eat breakfast<br>• Snack between meals<br>• Eat fried meat	Never/ once a week/ several times a week/ every day
**Section 4. Limitation of unhealthy products and unhealthy behaviors**	
**Do you try to limit your consumption of:**<br>• Sweets<br>• Fast food (e.g. hamburger, hot dog, pizza)<br>• Fried meat<br>• Sweetened beverages (e.g. coca-cola, pepsi, sprite, fanta, lemonade);<br>• Energy drinks (e.g. red bull)<br>• Alcohol in total	I do not limit/ I am looking for healthier alternative/ I try to eat/drink very rarely/ I do not eat/drink
• Do you try to limit the amount of salt in your food?<br>• Do you try to limit the amount of sugar in your food?<br>• Do you try to limit snacking between meals?<br>• Do you try to limit your food at night?	I do not limit/ I am looking for healthier alternative/ I try to eat/drink very rarely/ I do not practice it
